# Do Fluctuations in Environmental Regulations Inhibit Investment: Evidence from China

**DOI:** 10.3390/ijerph20032021

**Published:** 2023-01-22

**Authors:** Ming Che, Hongmei Wu, Yujia Li

**Affiliations:** 1 School of Economics, Xihua University, Chengdu 610039, China; 2 School of Economics, Sichuan University, Chengdu 610065, China

**Keywords:** environmental regulation fluctuations, expected profits, investment, dynamic panel, mediating effects model

## Abstract

The trade-off between the goals of promoting economic growth and protecting the ecological environment makes it possible for the government to constantly adjust the intensity of environmental regulation, leading to sharp fluctuations in environmental regulation in the short term. Fluctuations in environmental regulations may trigger concerns among firms and change their investment decisions. The theoretical model of corporate investment decision is used to analyze the inhibitory effect of environmental regulation fluctuations on investment through expected profits, which is empirically validated in this study by data from 255 Chinese prefecture-level cities. The results indicate that environmental regulation fluctuations reduce investors’ expected profits, which in turn inhibit investment. The heterogeneity analysis shows that environmental regulation fluctuations have no significant effect on investment in cities that are geographically closer to the provincial capital, while a greater inhibitory effect of it is revealed in other cities located further away. Therefore, this inhibitory effect should be weakened by reducing the intervention of administrative orders in environmental regulatory behavior, establishing environmental regulatory supervisory agencies, and taking into full consideration the public’s response to fluctuations in environmental regulation. This study can provide policy implications for optimizing government environmental regulation.

## 1. Introduction

Since their reform and opening up in 1978, China has achieved rapid economic growth. However, this has been accompanied by the pressure of environmental damage, which has gradually become a bottleneck limiting China’s sustainable economic development [1]. As a result, China’s central government has implemented a series of regulatory measures aimed at protecting the environment. However, legislation and policy formulation are only the first step in protecting the environment; the subsequent enforcement and policy implementation are the keys to achieving the goals. In China, it is up to each local government to implement relevant policies set by the central government, but fiscal decentralization has triggered fierce competition among local governments, and local protectionism has influenced the implementation of environmental regulations [2], as evidenced by the ability of local governments to suspend or restrict the implementation of central government policies [3,4,5]. Local governments in China are burdened with the dual goals of promoting economic growth and protecting the ecological environment. Under this goal constraint, local government officials have incentives to use their administrative power to influence the enforcement of environmental regulations. For example, when the climate of social opinion is more relaxed, local governments will choose a relatively weak intensity of environmental regulation for the purpose of promoting local economic development and tolerate some of the negative externalities of economic activities [6]; with the accumulation of negative externalities and increasing pressure from public opinion, local government officials choose to increase the intensity of environmental regulation when they believe that the situation has become more severe, thus making the intensity of environmental regulation fluctuate drastically in the short term.

The sharp fluctuations of environmental regulations in the short-term may cause concern to enterprises. This is because when regulations fluctuate, investors expect that environmental regulations may increase in the future. At that time, companies will be required to deal with pollutants more stringently, which will increase their costs; in addition, investors will not be able to recover their upfront investment when regulations rise due to the existence of sunk costs. Both factors ultimately lead to a decline in expected profits. Therefore, a rational investor may choose to “wait” when faced with this kind of uncertainty until the regulatory intensity stabilizes before making a decision. In other words, the uncertainty associated with regulatory fluctuations generates an “option value” for potential investors, resulting in a disincentive to potential investment behavior [7,8]. However, Engau and Hoffmann suggest that firms can better cope with environmental regulatory uncertainty if they participate in policy formulation and increase strategic flexibility [9]; their results show that regulatory uncertainty also only partially causes firms to delay strategic decisions. In China, however, contexts in which firms participate in policy making may not be common, which limits firms’ ability to respond to environmental regulation fluctuations. Therefore, our study aims to empirically investigate the actual effects of environmental regulation fluctuations on Chinese investment. For this purpose, we ask the following questions: (1) Does environmental regulation fluctuations inhibit investment in China? (2) Does and how do expected profits moderate the relationship between environmental regulation fluctuations and investment? (3) Does the effect of environmental regulation fluctuations on investment vary significantly across cities?

The importance of the topic of this paper is at least twofold. First, it provides empirical evidence on the investment effects of environmental regulatory fluctuations from an emerging market economy. Second, our results have important policy implications. If fluctuations in environmental regulations do lead to lower investment, then the government should focus on reducing the magnitude of regulatory fluctuations, guiding firms to form stable expectations, and maintaining investment consistency as a way to reconcile the tension between “promoting economic growth” and “protecting the ecological environment”.

Our study has three main contributions. First, while the established literature mostly analyzes the impact of environmental regulations on investment [6,10,11,12]. Instead, from an uncertainty perspective, we propose that environmental regulations can fluctuate significantly in the short run, and that such fluctuations can themselves have an impact on investment. Second, we emphasize the mechanistic role of investor expectations, and our results show that environmental regulatory fluctuations will lead to increased investor uncertainty about expected profits, reduced expected profits, and inhibited investment. Finally, in order to measure the environmental regulation fluctuations, we first apply the entropy assignment method to obtain a pollutant treatment rate indicator by combining the industrial soot treatment rate, industrial sulfur dioxide treatment rate and solid waste treatment rate indicators, and use this composite indicator to proxy for environmental regulation intensity. Then we take 5 years as the fluctuation cycle of environmental regulation, and the moving standard deviation of the composite index is used to represent the environmental regulation fluctuations. Using five years as a cycle length is more in line with the change of local governments and the length of economic planning in China; when local government officials change or new economic plans are introduced, the strength of environmental policies implemented in each region will change and environmental regulations will fluctuate accordingly.

The rest of this paper is structured as follows. Section 2 reviews the relevant literature. Section 3 describes the theoretical link between environmental regulation fluctuations, investor expectations, and investment. Section 4 describes the empirical strategy. Section 5 discusses the empirical results. Section 6 concludes.

## 2. Literature Review

The design and implementation of environmental regulation policies is a dynamic process in which the drafters of the regulations need to continuously adjust and optimize [13]. By the time the environmental regulation policy is formally released, the effectiveness of the policy implementation, however, depends on the implementation by local governments, which in turn depends largely on the intentions of local governments [2,14,15]. However, local governments face a trade-off between promoting economic growth and protecting the environment, which makes the implementation of environmental regulations fluctuate in the long-term tension between the two goals [16]. In addition, Zhang et al. summarized other reasons for the large fluctuations in environmental regulation in China, involving incomplete scope of penalties, the fact that specific penalties are determined by local regulators, and complex and lengthy enforcement applications [6]. Several studies have been conducted to analyze the impact of regulatory uncertainty on firms’ investment decisions.

Hoffmann et al. defined regulatory uncertainty as “the perceived inability of individuals to predict the future state of the regulatory environment” [17], a state that can limit firms’ ability to make decisions and choose appropriate responses [18]. Intuitively, the value of a firm’s waiting option to invest increases if the final outcome of environmental regulatory policies is uncertain and potentially harmful to the firm [7,8,19]. More accurate information enables firms to better assess the returns and risks of investment projects [20], while uncertainty may make firms tend to delay investments [21]. This argument is supported by several empirical findings. Rogge et al. study the inherent uncertainty of climate policy and find that it makes it impossible for firms to make accurate investment plans and weakens the willingness for innovative investments [22]. Barradale treated uncertain climate policy as an external risk and stated that investors tend to delay their investments to hedge the risk [23]. In addition, Ritzenhofen and Spinler studied the impact of feed-in-tariffs (FITs) on investments in renewable energy sources and find that when FIT levels are close to market prices, environmental regulatory uncertainty can make firms likely to delay or reduce their investments [24]. Xie et al. examined the impact of formal and informal environmental regulatory fluctuations on firms’ investment in technological innovation and find that both types of regulatory fluctuations stifle firms’ willingness to innovate and lead to a reduction in investment [13]. Azqueta-Gavaldón et al. calculated economic uncertainty indicators that capture changes in regulation and find that domestic regulatory uncertainty components have considerable effects on investment [25].

However, other studies take a different view, arguing that regulatory uncertainty does not necessarily delay or inhibit firm investment, and may even have a catalytic effect on investment. The theoretical basis for this type of research is mainly the resource-based view of the firm [19,26,27] and the basic principles of institutional theory. Hoffmann et al. suggested that in the face of regulatory uncertainty, firms may be motivated to invest in order to secure competitive resources, exploit complementary resources, and mitigate institutional pressures [28]. For example, Aragon-Correa and Sharma used a theoretical model to show that when the environment is unstable, firms seek new developments to gain competitive advantages [29]. Carrera et al. analyze a survey of 33 firms in Argentina and find that uncertainty in the regulatory and business environment causes firms to increase their investments in portfolio expansion to diversify risk [30]. Engau and Hoffman showed, based on worldwide survey data, that firms respond to regulatory uncertainty by engaging in policy-making and increasing strategic flexibility, and that regulatory uncertainty actually only partially causes firms to delay investment decisions [9]. Alternatively, some studies have found no statistically significant relationship between regulatory uncertainty and firm investment [19].

Taken together, the results of the above studies reveal that the impact of environmental regulatory fluctuations on investment depends on the firm’s response, which in turn is related to the firm’s tolerance and perception of uncertainty. Prior research has shown that there are differences in individuals’ perceptions and tolerance of uncertainty [19,31], which leads to differences in firms’ investment decisions. Therefore, in this study, we adopt a model of firms’ investment decisions to analyze the different responses of firms in the face of environmental regulation fluctuations and investigate whether environmental regulation fluctuations have a inhibitory effect on investment in China using data from 255 prefecture-level cities.

## 3. Theoretical Analysis

Drawing on Handley and Limão’s modeling framework [32], we assume that there is a large number of monopolistically competitive firms in the market and that the firms produce only one variety of differentiated product, *m*. The product demand curve is assumed to be $q_{m}=P^{\sigma -1}p_{m}^{-\sigma }$, where σ>1, and σ is the fixed elasticity of substitution between heterogeneous goods in the consumer utility function. *p_m_* is the price of product *m* and $P={\left[\int_{0}^{1}{\left(p_{m}\right)}^{1-\sigma }dm\right]}^{\frac{1}{1-\sigma }}$ is the CES price index. We assume that there are three different kinds of environmental regulation strengths, *i*, i=0,1,2. For different i, the environmental regulation costs τ paid by enterprises are different, $\tau_{2}>\tau_{1}>\tau_{0}$. Assuming that the current environmental regulation intensity is i=1 and the probability that the environmental regulation intensity fluctuates in the future is γ. The variation of environmental regulation intensity across periods follows a Markov process $\Lambda \left(\tau_{i},\gamma \right)$. Environmental regulation incurs an ad valorem environmental regulation cost to the firms, such as coping with unannounced government inspections or trying to meet environmental standards set by the government, and the cost is recorded as $\tau_{i}-1$, and $\tau_{i}>1$. The cost of environmental regulation is proportional to the price *p_m_*, so that the actual revenue received by the firm after selling the good is $\frac{p_{m}}{\tau_{i}}$. In addition, when a firm sells a product, it has to bear a certain sales cost, which is recorded as $\delta_{m}-1$, $\delta_{m}\geq 1$. Therefore, the firm’s revenue per unit of product sold is $\frac{p_{m}}{\tau_{i}}-\delta_{m}c_{m}$, where $c_{m}$ is the marginal cost. Firms maximize profits by choosing the selling price of their products under the assumption of rational expectations. Therefore, the equilibrium price of product *m* is $p_{m}=\tau_{i}\delta_{m}c_{m}$. An investor who wants to enter the market has to pay the sunk costs *S*. When there are fluctuations in environmental regulations, that is, the intensity of the environmental regulations to which a firm is subject may change, investors may choose to wait or pay the sunk cost and enter the market. Investors determine whether to enter the market by observing the operating conditions of their peers in the previous period, the intensity of environmental regulations in the current period, and the model parameters at the beginning of each period. After entering the market, the survival probability of the firm is α for every period.

Here we consider two scenarios: one without environmental regulation fluctuations and the other with environmental regulation fluctuations. When there is no environmental regulation fluctuation, by solving the profit maximization problem and the market entry condition $S\left(1-\alpha \right)=\pi \left(\tau_{i},c_{m}\right)$, the profit of the firm and the threshold value of marginal cost for market entry can be obtained as,

(1) $$\pi \left(\tau_{i},c_{m}\right)=\left(\frac{p_{m}}{\tau_{i}}-\delta_{m}c_{m}\right)q_{m}=b\tau_{i}^{-\sigma }c_{m}^{1-\sigma }\delta_{m}^{1-\sigma }$$

(2) $$c_{m}^{D}={\left[\frac{b\tau_{i}^{-\sigma }\delta_{m}^{1-\delta }}{S\left(1-\alpha \right)}\right]}^{\frac{1}{\sigma -1}}$$

where $b=\sigma^{-\sigma }{\left[P\left(\sigma -1\right)\right]}^{\sigma -1}$.

When the intensity of environmental regulation may fluctuate, investors can choose to enter the market or continue to wait, and there exists a threshold marginal cost $c_{m}^{U}$ making them indifferent between the two decisions, that is,

(3) $$\Pi_{e}\left(c_{m}^{U},\bar{\tau }\right)-S=\Pi_{w}\left(c_{m}^{U},\bar{\tau }\right)$$

where $\Pi_{e}$ and $\Pi_{w}$ denote the expected profits of investors for entering the market and continuing to wait in the current period, respectively. When the environmental regulation intensity $\tau_{i}$ is lower than $\bar{\tau }$, investors will choose to enter the market because of the lower cost. If the intensity of environmental regulation fluctuates with probability γ, we denote the new intensity of environmental regulation as $\tau_{-1}$ and $\tau_{-1}\in \left[\tau_{0},\tau_{2}\right]$. The assumption is that investors expect the intensity of environmental regulations to become weaker with probability φ and stronger with probability 1−φ. Therefore,

(4) $$\Pi_{e}\left(c_{m}^{U},\tau_{1}\right)=\pi \left(c_{m}^{U},\tau_{1}\right)+\alpha \left[\left(1-\gamma \right)\Pi_{e}\left(c_{m}^{U},\tau_{1}\right)+\gamma E\Pi_{e}\left(c_{m}^{U},\tau_{-1}\right)\right]$$

Equation (4) indicates that investors who choose to enter the market in the current period will earn $\pi \left(c_{m}^{U},\tau_{1}\right)\;$ in the current period. If the firm continues to survive with probability α, the profit is $\Pi_{e}\left(c_{m}^{U},\tau_{1}\right)$ when the intensity of regulation is constant, and the expected profit is $E\Pi_{e}\left(c_{m}^{U},\tau_{-1}\right)$ when the environmental regulation fluctuates.

(5) $$\Pi_{w}\left(c_{m}^{U}\right)=0+\alpha \left[\begin{array}{c} \left(1-\gamma \right)\Pi_{w}\left(c_{m}^{U}\right)+\gamma \left(1-\varphi \left(\bar{\tau }\right)\right)\Pi_{w}\left(c_{m}^{U}\right)+\\ \gamma \varphi \left(\bar{\tau }\right)\left(E\left(\Pi_{e}\left(c_{m}^{U},\tau_{-1}\right)|\tau_{-1}\leq \bar{\tau }\right)-S\right) \end{array}\right]$$

Equation (5) indicates that investors continue to wait in the current period, the profit is zero. If the firm survives with probability α, they will choose to maintain wait-and-see in two scenarios, either when the intensity of the environmental regulation does not fluctuate, or when it fluctuates and investors believe it will become stronger. The profit remains $\Pi_{w}\left(c_{m}^{U}\right)$ in both scenarios. In addition, when the intensity of environmental regulation fluctuates and investors believe that it will become weaker, that is, $\tau_{-1}\leq \bar{\tau }$, they will pay the sunk cost *S*, enter the market, and earn the expected profit $E\left(\Pi_{e}\left(c_{m}^{U},\tau_{-1}\right)|\tau_{-1}\leq \bar{\tau }\right)$. Therefore, we can obtain the following equation relating $c_{m}^{U}$ and $c_{m}^{D}$,

(6) $$c_{m}^{D}U\left(\omega ,\gamma \right)=c_{m}^{U}$$

(7) $$U\left(\omega ,\gamma \right)={\left(\frac{1-\alpha \left(1-\gamma \omega \right)}{1-\alpha \left(1-\gamma \right)}\right)}^{\frac{1}{\sigma -1}}$$

where $\omega =\varphi \left(\tau_{1}\right)+\frac{\left(1-\varphi \left(\tau_{1}\right)\right)E\left(\tau_{-1}^{-\sigma }|\tau_{-1}\geq \bar{\tau }\right)}{\tau_{1}^{-\sigma }}$. We know from $\frac{\partial c_{m}^{U}}{\partial \gamma }<0$ that when the likelihood of fluctuations in environmental regulations is higher, that is, the stronger the fluctuations in environmental regulations, the higher the production cost for investors to enter the market, the higher the entry barrier, and the more pronounced the inhibitory effect on investment.

## 4. Empirical Strategy

### 4.1. Baseline Regression Model

When we investigating the impact of environmental regulation volatility on investment, there may be endogeneity issues that investment may be driven by an unobservable component that affects regulation volatility, and this unobservable component can be captured by the lagged term of investment, OLS estimation as well as traditional panel estimation methods cannot obtain valid estimates. Therefore, we include the first-order lagged term of investment in the equation. In determining the optimal lag terms, Glen et al. and Gschwandtner argue that two lags are sufficient to capture persistence, and indeed [33,34], we have tried to include the second-order lag term for investment in the equation, but it did not give us more information because it did not have a significant effect on current investment and the regression coefficients were difficult to interpret in theoretical terms, and the coefficient estimates of the remaining variables did not have much difference compared to the baseline model. Therefore, we believe that the first-order lagged term of investment included in the equation is sufficient. We use the dynamic panel GMM estimator to deal with the above endogeneity issues [35], where the baseline regression model is shown below:

(8) $$IN_{it}=\beta_{0}+\beta_{1}IN_{it-1}+\beta_{2}VF_{it}+\beta_{3}X_{it}+\varepsilon_{it}$$

where *IN* represents investment; *VF* denotes the fluctuation of environmental regulations and is the core explanatory variable of the equation; *X* represents the control variables; ε is the residual term, $\beta_{0}$ is the constant term, and the subscripts *i* and *t* represent city and time, respectively. In the theoretical analysis, we infer that the stronger the fluctuations of environmental regulation, the more pronounced its inhibitory effect on investment will be. To verify this point, we employ $\beta_{2}$ to measure the impact of environmental regulation fluctuation on investment. If $\beta_{2}$ is statistically significantly negative, then our inference is supported by the data that environmental regulation fluctuations have an inhibitory effect on investment.

### 4.2. Mechanism Testing Model

Based on the analysis of the previous theoretical model, it is clear that environmental regulation fluctuations influence firms’ investment decisions by affecting their expected operating profits. This hypothesis is also supported by some theories. According to the option value theory, it pays for investors to wait when exposed to uncertainty. In this case, the uncertainty provides a “waiting option value” for the investor. That is, if the investor chooses to invest right away, then part of the investment cannot be recovered due to sunk costs when the economic environment deteriorates, which results in a potential loss for the investor. If the investor chooses to wait, the potential return when the economic environment gets better becomes a potential loss for this investor at the moment. Therefore, investors will choose to invest only if the expected return on investment under uncertainty is higher than the sunk cost; otherwise, rational investors will postpone investing [7,8]. In addition, there are also studies analyzing the impact of uncertainty on firms’ investment decisions and the mechanisms involved in it based on prospect theory [36]. A recent related investigation derives from Xie et al. [13]. They investigate the impact of environmental regulation regulations on technological innovation, an important investment, using data from 36 Organization for Economic Cooperation and Development countries from 2013–2018. The study shows that environmental regulation fluctuations increase the unpredictable risk for investors and significantly inhibit the willingness to innovate activities. Combining the above theoretical analysis and empirical results, we infer that when exposed to fluctuations in environmental regulations, investors may delay or abandon their investments due to concerns about expected profits. The following mediating effects model is therefore chosen to test the plausibility of this mechanism.

(9) $$IN_{it}=\alpha_{0}+\alpha_{1}IN_{it-1}+\alpha_{2}VF_{it}+\alpha_{3}X_{it}^{*}+\varepsilon_{it}$$

(10) $$EIN_{it}=\varphi_{0}+\varphi_{1}EIN_{it-1}+\varphi_{2}VF_{it}+\varphi_{3}X_{it}^{*}+\varepsilon_{it}$$

(11) $$IN_{it}=\gamma_{0}+\gamma_{1}IN_{it-1}+\gamma_{2}EIN_{it}+\gamma_{3}VF_{it}+\gamma_{4}X_{it}^{*}+\varepsilon_{it}$$

In Equations (9)–(11), *EIN* represents investors’ expected profits, $X_{it}^{*}$ represents the control variables, and the rest of the variables remains unchanged. The first step in conducting the mechanism test is to run a regression of Equation (11). It should be noted that although the variables in Equations (8) and (9) are the same, we use different coefficient symbols in the two equations for the following two reasons: (a) The implications of these two equations are different in that Equation (8) represents our baseline regression using the full sample, whereas Equation (9) is the first step in our mediating effects model. (b) The two equations use different sample sizes. In the baseline regression represented by Equation (8), we use a sample including 255 prefecture-level cities, while in the mediating effects model, Equation (9) uses data from only 146 prefecture-level cities because some cities do not provide data on the amount of foreign direct investment contracts. $\alpha_{2}$ in Equation (9) represents the total effect of environmental regulation fluctuations on investment and its significance is crucial to the overall mechanism test. If $\alpha_{2}$ is not statistically significant, then we cannot ensure that environmental regulation fluctuations have a significant effect on investment and the mechanism test is not necessary to be proceed [37]. If $\alpha_{2}$ passes the significance test, we then run regression analysis on Equations (10) and (11), and if both $\varphi_{2}$ and $\gamma_{2}$ are statistically significant, the mediation effect holds, that is, environmental regulation fluctuations affect investment through firms expected operating profits. At this point, if $\gamma_{3}$ is statistically insignificant, then environmental regulation fluctuations affect investment only through firms expected operating profits, and there are no other paths of action. Conversely, if $\gamma_{3}$ is statistically significant, it suggests that environmental regulation fluctuations can affect investment through channels other than expected operating profit.

### 4.3. Variable Selection

#### 4.3.1. Environmental Regulation Fluctuations

**Figure 1:**
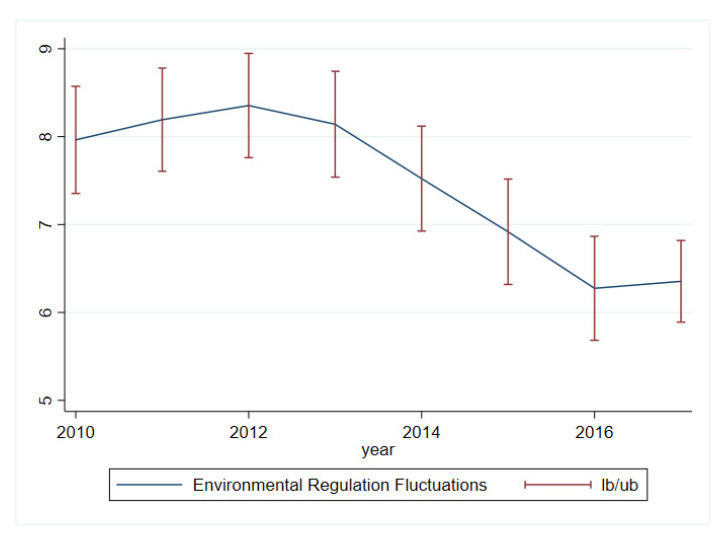
Environmental regulation fluctuations in China. Note: The sample is from 2010 to 2017.

We begin by measuring the intensity of environmental regulation when measuring environmental regulation fluctuations. Among the existing research results, scholars have tried to measure the intensity of environmental regulation in terms of the emission intensity of industrial pollutants [38], pollution discharge fees [39], the operating cost of industrial waste gas treatment equipment [1], and environmental protection investment [40], and so forth. Given the availability of data, we use the entropy method to assign weights to industrial soot treatment rate, industrial sulfur dioxide treatment rate, and solid waste treatment rate to obtain a composite pollutant treatment rate indicator, and consider it as a proxy for the intensity of environmental regulation. The weights of industrial soot treatment rate, industrial sulfur dioxide treatment rate and solid waste treatment rate are 0.149, 0.614 and 0.237, respectively. Referring to Braun and Larrain and, we use the moving standard deviation of the intensity of environmental regulation for a given period to characterize the state of environmental regulation fluctuations [41]. We use 5 years as the period to measure environmental regulation fluctuations, that is, we capture the fluctuation of environmental regulation in year *t* by calculating the moving standard deviation of the intensity of environmental regulation from year *t* − 5 to year *t* − 1, with the larger the standard deviation the stronger the fluctuation. This indicator responds both to fluctuations in the intensity of environmental regulation over time and to trends in such fluctuations over time. The reason for choosing a 5-year moving standard deviation is that this length of time encompasses the cycle of official turnover and economic planning for local governments in China; when officials change and new plans are made, local policies will change accordingly and the intensity of environmental regulations may change. Figure 1 illustrates the environmental regulation fluctuations of Chinese prefecture-level cities during 2010–2017; the solid line in the figure is the median, and the dashed line segment is the range of variation of environmental regulation fluctuations for all cities. Overall, China’s environmental regulation fluctuations show an upward and then downward trend during 2010–2017, with environmental regulation fluctuations gradually increasing during 2010–2012 and decreasing thereafter, but rebounding slightly in 2017.

#### 4.3.2. Investment

The established literature usually uses indicators such as social fixed asset investment, foreign direct investment (FDI), and investment in state-controlled enterprises to represent investment, but social fixed asset investment and investment in state-controlled enterprises are not applicable to the analysis framework of this paper. The reason is that social fixed asset investment includes not only enterprise investment, but also investment in fixed assets of institutions and administrative units, which are not profit-oriented and also have certain counter-cyclical characteristics (the “quadrillion plan” between 2008 and 2010 is a good example); state-controlled enterprises have policy burdens such as expanding employment in the process of operation, and their investment behavior does not fully reflect the changes in their expected operating profits. On the other hand, foreign direct investment is characterized by a high sunk cost and very sensitive to the investment environment, which is in line with the relevant assumptions of the theoretical research framework of this paper, and its data availability is better, so we chose the logarithm of the actual amount of foreign investment utilized in the year to reflect the investment level.

#### 4.3.3. Expected Profit

In this paper, we choose the logarithmic value of the FDI contract amount in the current year to reflect the expected profit level. Since the contract amount of FDI is not the final actual amount of utilized foreign capital, but the investment plan estimated by foreign investors based on their expected business conditions after comprehensive analysis of the investment environment, the amount of FDI contract is directly related to the business expectation of the invested area, which can reflect the expected business profit of foreign investors to a degree. Therefore, this paper chooses the logarithm of the amount of foreign direct investment contracts in the current year to represent the expected profit of investors. 

#### 4.3.4. Control Variables

In addition to the above variables, we also selected control variables from the following five aspects. A higher level of economic development in a region implies that consumers have stronger purchasing power and are more attractive to FDI, so we included the level of economic development expressed as the logarithm of GDP per capita in the equation. A region that is more open to the world will be more inclusive of FDI, so we controlled for the degree of openness of each city, expressed as a share of total imports and exports in GDP. For foreign investors, the availability of capital and the wage level of workers are two important factors. The higher the availability of capital and the lower the wage level, the more attractive cities are for FDI, so we added the size of the financial market and the average wage level to the equation, which are expressed as the logarithm of the balances of loans from financial institutions and the logarithm of the average wage of workers, respectively. In addition, FDI in China is mainly concentrated in the industrial sector, and the industrial structure of each city also has an impact on the investment decisions of foreign investors, so we control the share of added value of the secondary industry in GDP. All of the above variables exclude the effect of price changes.

#### 4.3.5. Data Sources and Descriptive Statistics

As of 2019, there are 293 prefectural-level cities in China, and excluding some cities with serious missing data, the sample in the baseline regression of this paper includes 255 prefectural-level cities, and the time interval of the original sample is 2005–2017. When measuring the environmental regulation fluctuations, a period of 5 years is used, so the actual time interval in the regression is 2010–2017. Due to the poor availability of data on the amount of foreign direct investment contracts in each city, the sample of 146 prefecture-level cities is included in the mechanism test. The data were obtained from the China City Statistical Yearbook, the China Regional Statistical Yearbook, the CEIC database, and some provincial and prefecture-level city statistical yearbooks. Table 1 shows the descriptive statistics of the data.

## 5. Empirical Results

### 5.1. Baseline Regression

Since the regression results of the dynamic panel GMM estimator are more sensitive to the model settings, we gradually add control variables to the baseline regression model to observe the dynamic changes of the regression results, and combine the results of Arellano–Bond test and Hansen J test to judge the reasonableness of the model settings. The results are shown in Table 2.

As can be seen in Table 2, the results of the Arellano–Bond test, in which control variables are continuously added, all indicate that there is first-order autocorrelation in the residuals of the regression equation, but not second-order autocorrelation, and the regression results of each column also pass the Hansen J test at the 5% significance level. Together with the fact that the regression coefficients and significance of all explanatory variables, except GDP per capita, did not change significantly, the regression model set up in this paper and the dynamic panel GMM estimation results can be considered reasonable.

The regression coefficient of environmental regulation fluctuation is always significantly negative when the control variables are continuously added, indicating that environmental regulation fluctuation will inhibit foreign investors from investing in the local area to some extent. In terms of control variables, the rise in per capita wage increases the cost of employing labor locally for foreign investors, which in turn decreases local FDI; while the rise in financial market size, openness level and industrial structure indicators significantly increase FDI.

### 5.2. Robustness Test

#### 5.2.1. Alternative Calculation Cycles for Environmental Regulation Fluctuations

It is clear from the benchmark regression that environmental regulation fluctuations would inhibit FDI. In the baseline regression, the period of environmental regulation fluctuations is measured to be 5 years, but investors may make investment decisions based on different information sets because of the different maturities of investment projects and differences in investors’ sensitivity to risk. Therefore, we test the robustness of the baseline results by narrowing and enlarging the calculation cycle of environmental regulation fluctuations, respectively. Since the average tenure of local officials in China is 4–6 years, we will test the inhibitory effects of environmental regulation fluctuations on investment with a 4-year and 6-year period, respectively. We note *std4* and *std6* as the environmental regulation fluctuations calculated with a 4-year and 6-year cycle. The results of columns (1) and (2) in Table 3 show that environmental regulation fluctuations have a dampening effect on FDI whether the calculation period is shortened or lengthened, and the regression coefficients are not significantly different from the baseline results.

#### 5.2.2. Alternative Measures of Environmental Regulation Fluctuations

In the baseline regression, the environmental regulation intensity index is constructed based on the treatment of pollutants in each city. To test the robustness of the benchmark results, we construct environmental regulation intensity indicators here from the perspective of emission intensity per unit of output. We divide the industrial wastewater, industrial sulfur dioxide and industrial smoke emissions of each city by the added value of the secondary industry in that year to calculate the emission intensity of each pollutant per unit of output, and then add them up to obtain the environmental regulation intensity index through the entropy method. The weights of industrial wastewater emissions per unit output value, industrial sulfur dioxide emissions per unit output value and industrial smoke emissions per unit output value calculated by the entropy method are 0.268, 0.300 and 0.432, respectively. Finally, we calculate the 5-year moving standard deviation of this index to obtain the environmental regulation fluctuation index. As can be seen in column (3) of Table 3, the conclusion that environmental regulation fluctuations inhibit local FDI still holds, despite the change in measurement, and the results in the benchmark regression are robust.

### 5.3. Mechanism Test

To test whether environmental regulation fluctuations affect investment through firms’ expectations, we conducted a mechanism test using the mediating model. The results of the mechanism test are presented in columns (1) to (3) in Table 4. The results in column (1) show that the regression coefficients of environmental regulation fluctuations are still statistically significant even when the sample size is reduced to 146 cities, which indicates the robustness of the baseline regression results.

The regression coefficient of environmental regulation fluctuations in column (2) is significantly negative, indicating that environmental regulation fluctuations reduce the expected profits of foreign investors in the local area. The regression coefficient of expected profits in column (3) is significantly positive, which indicates that the mechanism in the previous theoretical analysis holds, that is, environmental regulation fluctuations will inhibit investment by reducing investors’ expected profits.

### 5.4. Heterogeneity Analysis

Cities in China have different administrative hierarchies. The higher administrative hierarchy of provincial capitals enables them to gather various scarce resources more easily, which makes them ahead of other cities in the province in terms of capital, technology, talent and infrastructure. A higher gathering of resources is often accompanied by developed transportation, smooth information collection channels and convenient access to resources, which helps reduce the sunk cost of FDI and thus reduces the sensitivity of FDI to environmental regulation fluctuations. The cities closer to the provincial capital city are more radiated by the high-quality resources of the provincial capital city, the sunk cost of FDI is lower, and the inhibiting effect of environmental regulation fluctuation on FDI is relatively smaller; while for the cities farther away from the provincial capital city, because they are less radiated by the resources of the provincial capital city, the sunk cost of FDI is higher, and the inhibiting effect of environmental regulation fluctuation on FDI will be greater.

To test this inference, we divided the total sample into two subsamples based on the average distance of each prefecture-level city from the capital city of the province. Columns (1) and (2) in Table 4 show the regression results for the prefecture-level cities whose distances to the provincial capital city are greater and less than the mean, respectively. The results show that the inhibitory effect of environmental regulation fluctuations on FDI is not statistically significant in the prefecture-level cities that are relatively close to the specific provincial capital city, while the inhibitory effect of environmental regulation fluctuations on FDI is relatively larger in the prefecture-level cities that are far from the provincial capital city.

## 6. Conclusions

In this paper, we studied the inhibitory effects of environmental regulation fluctuations on investment. We used a firm investment decision model to explore the impact of environmental regulation fluctuations on investment, and the model results show that environmental regulation fluctuations inhibit the expansion of investment by raising entry barriers and lowering investors’ profit expectations. Further, we used panel data of 255 prefecture-level cities in China from 2005–2017 to validate the findings of the theoretical model using a dynamic panel GMM estimator and tested the mechanism with the help of a mediating effects model. The results found that the fluctuation of environmental regulations in each city inhibits the scale of investment. This conclusion still holds after the relevant robustness tests. In addition, the heterogeneity analysis in this paper shows that the inhibitory effect of environmental regulation fluctuations on investment is insignificant in prefecture-level cities that are relatively close to the provincial capital city, while it is relatively larger in cities that are far from the provincial capital city. 

In contrast to much of the established literature analyzing the effects of environmental regulation on investment [6,10,11,12], we focused on the effects of fluctuations in environmental regulation on investment. Our results imply that even if the government wants to stimulate investment by rapidly reducing the intensity of environmental regulations, it may not be able to achieve its desired outcomes. This is because sharp fluctuations in the intensity of regulation in the short-term can itself cause investors to lose confidence and choose to wait and see, preventing a rapid increase in investment. In addition, the weakening intensity of regulation may trigger new environmental problems. In this way, a stable intensity of environmental regulation plays an important role in alleviating the contradiction between “promoting economic growth” and “protecting the ecological environment”. The research in this paper has some policy implications.

First, the intervention of administrative orders in the government’s environmental regulation should be reduced to ensure that all environmental regulation policies are formulated and environmental regulation acts are carried out legally and compliantly. Reducing the direct or indirect intervention of administrative orders in the formulation of environmental regulation to ensure that environmental regulation policy formulation reflects its characteristics of improving the efficiency of resource allocation and compensating for the defects of the market operation mechanism. The direct or indirect intervention of administrative orders in the process of environmental regulation implementation should also be reduced, and the process of environmental regulation enforcement should be strictly regulated. By strengthening the relative independence of environmental regulation sectors, we can reduce the possibility of fluctuations in environmental regulation due to administrative power, thereby promoting the steady increase of investments.

Second, we should establish environmental regulation supervisory agencies to reduce the possibility of environmental regulation fluctuations through before, during and after supervision over environmental regulation sectors. Before the environmental regulation policy is formulated, the agencies should monitor whether there are economic agents seeking rent from the environmental regulation sector for individual interests to ensure the fairness and reasonableness of the policy. During the process of environmental regulation policy formulation, the agencies shall monitor whether the environmental regulation department formulates environmental regulation policy in strict accordance with the legal procedures and within the scope of authority and responsibility stipulated in the law, and shall severely punish those who fail to formulate policy in accordance with the requirements of the law. After the environmental regulation policy is formulated, the agencies should monitor whether the environmental regulation department implements the environmental regulation acts according to the policy, and hold the responsible persons and departments accountable for the acts of not exercising the environmental regulation as required.

Third, when carrying out environmental regulation reforms, the public’s reaction to environmental regulation fluctuations should be fully considered. Policymakers should be aware that although environmental regulation reform will reduce the burden of business operations, frequent fluctuations in environmental regulation will affect the expected profits of enterprises and inhibit investments in expansion. Therefore, in the process of deepening market-oriented reforms, the government should ensure that environmental regulation reforms are carried out smoothly and not too aggressively, and create a stable investment environment for enterprises by reducing the magnitude of environmental regulation fluctuations, so as to prevent enterprises from postponing their investments due to the changing investment environment.

## Figures and Tables

**Table 1:** Descriptive statistics of variables.

Variables	Obs	Mean	Std. Dev.	Min	Max	Skewness	Kurtosis	JB-Test
Environmental Regulation Fluctuations (*VF*)	2040	7.465	4.777	0.295	28.084	1.030	3.923	433.3 (0)
Foreign Direct Investment (*IN*)	2040	11.299	2.658	0	19.599	−2.408	10.999	7411 (0)
Foreign direct investment contract amount (*EIN*)	1168	11.241	2.650	0	17.086	−2.259	10.423	3675 (0)
Loan balance of financial institutions (Loan)	2040	15.923	1.044	13.301	19.485	0.777	3.322	214.3 (0)
Average wage (Wage)	2040	10.4	0.277	8.119	12.898	0.167	9.359	3448 (0)
GDP per capita (Pgdp)	2040	10.308	0.592	8.197	12.019	−0.027	2.880	1.481 (0.48)
Total Import and Export (Trade)	2040	13.510	2.030	1.512	19.351	−0.198	3.997	97.99 (0)
The share of added value of secondary industry in GDP (Industry)	2040	49.670	9.611	14.832	82.240	−0.092	3.806	58.18 (0)

Note: Obs is the sample size, Mean is the mean value, Std. Dev. is the standard deviation, Min is the minimum value, Max is the maximum value, JB-test is the Jarque–Bera asymptotic test, and the probability that the variable follows a normal distribution is in parentheses.

**Table 2:** Baseline regression results.

	(1)	(2)	(3)	(4)	(5)
	*IN*	*IN*	*IN*	*IN*	*IN*
*IN_t_*_−1_	0.497 ***	0.519 ***	0.521 ***	0.514 ***	0.502 ***
	(8.184)	(7.495)	(7.870)	(7.430)	(7.333)
*VF*	−0.029 *	−0.043 **	−0.041 **	−0.044 **	−0.045 **
	(−1.778)	(−2.208)	(−2.158)	(−2.283)	(−2.358)
Wage		−1.728 ***	−1.987 ***	−1.726 ***	−1.570 ***
		(−5.329)	(−5.578)	(−4.579)	(−4.179)
Loan		0.863 ***	0.751 ***	0.609 ***	0.646 ***
		(6.204)	(5.527)	(4.193)	(4.429)
Pgdp			0.453 **	0.308	0.223
			(2.419)	(1.541)	(1.141)
Trade				0.111 **	0.107 **
				(1.976)	(1.973)
Industry					0.012 *
					(1.673)
Constant term	5.945 ***	10.000 ***	9.762 ***	9.427 ***	7.683 ***
	(8.323)	(3.858)	(3.779)	(3.683)	(2.920)
AR(1)	0.000	0.000	0.000	0.000	0.000
AR(2)	0.546	0.621	0.619	0.596	0.602
Hansen J test	0.098	0.225	0.233	0.532	0.802
Total sample size	1785	1785	1785	1785	1785
Number of Cities	255	255	255	255	255

Note: ***, **, * indicate that the regression coefficients are statistically significant at the 1%, 5%, and 10% levels, respectively, and the z-statistic is in parentheses.

**Table 3:** Robustness tests.

	(1)	(2)	(3)
	*IN*	*IN*	*IN*
*IN_t_*_−1_	0.405 ***	0.549 ***	0.429 **
	(2.953)	(3.061)	(2.166)
std4r	−0.050 *		
	(−1.868)		
std6r		−0.034 **	
		(−1.974)	
*VF*			−0.028 *
			(−1.874)
Wage	−1.802 ***	−0.705	−0.060
	(−4.333)	(−1.369)	(−0.074)
Loan	0.740 ***	0.151	0.999 ***
	(4.422)	(0.643)	(3.549)
Pgdp	0.229	−0.092	−2.139 *
	(1.141)	(−0.314)	(−1.954)
Trade	0.154 *	0.369 **	0.327 **
	(1.825)	(2.234)	(1.989)
Industry	0.017 **	0.011	0.030 **
	(2.375)	(1.167)	(2.361)
Constant term	8.788 ***	5.819 **	7.421 **
	(3.089)	(2.373)	(2.568)
AR(1)	0.003	0.005	0.003
AR(2)	0.886	0.544	0.701
Hansen J test	0.664	0.369	0.502
Total sample size	1785	1785	1785
Number of Cities	255	255	255

Note: ***, **, * indicate that the regression coefficients are statistically significant at the 1%, 5%, and 10% levels, respectively, and the z-statistic is in parentheses.

**Table 4:** Mechanism test and heterogeneity analysis.

	(1)	(2)	(3)	(4)	(5)
	*IN*	*EIN*	*IN*	*IN*	*IN*
*IN_t_*_−1_	0.496 ***		0.506 ***	0.480 ***	0.568 ***
	(5.678)		(5.510)	(5.008)	(5.988)
*EIN_t_*_−1_		0.039			
		(0.302)			
*EIN*			0.282 ***		
			(3.680)		
*VF*	−0.055 **	−0.089 *	−0.051 *	−0.079 **	0.011
	(−1.987)	(−1.852)	(−1.668)	(−2.403)	(0.666)
Wage	−1.407 ***	2.482	−1.780 *	−1.464 ***	−1.685 ***
	(−3.214)	(1.277)	(−1.842)	(−2.586)	(−3.645)
Loan	0.694 ***	1.218 **	0.785 *	0.718 **	0.571 ***
	(3.950)	(2.106)	(1.811)	(2.255)	(4.502)
Pgdp	0.013	−5.580	0.516	−0.243	0.597 *
	(0.045)	(−1.621)	(0.701)	(−0.876)	(1.920)
Trade	0.138 **	1.063 **	−0.310	0.161	0.044
	(2.031)	(2.290)	(−0.741)	(1.626)	(0.848)
Industry	0.007	−0.030	0.017	−0.003	0.024 **
	(0.855)	(−0.485)	(1.614)	(−0.242)	(2.365)
Constant term	7.345 **	10.589	6.868 **	10.733 ***	5.319 *
	(2.233)	(0.950)	(1.989)	(2.882)	(1.812)
AR(1)	0.001	0.000	0.001	0.001	0.007
AR(2)	0.754	0.548	0.728	0.716	0.757
Hansen J test	0.732	0.412	0.768	0.797	0.948
Total sample size	1022	1022	1022	826	959
Number of Cities	146	146	146	118	137

Note: ***, **, * indicate that the regression coefficients are statistically significant at the 1%, 5%, and 10% levels, respectively, and the z-statistic is in parentheses.

## Data Availability

The data that support the findings of this study are available from the corresponding author upon reasonable request.
